# High Canonical Wnt/β-Catenin Activity Sensitizes Murine Hematopoietic Stem and Progenitor Cells to DNA Damage

**DOI:** 10.1007/s12015-019-09930-2

**Published:** 2019-12-03

**Authors:** Yiting Wang, Hui Cui, Si Tao, Ting Zeng, Jianying Wu, Zhendong Tao, Liu Zhang, Bing Zou, Zhiyang Chen, George B. Garside, Duozhuang Tang

**Affiliations:** 1 Department of Hematology, The Second Affiliated Hospital of Nanchang University, Min-De Road. 1, Nanchang City, 330006 Jiangxi Province China; 2 Jiangxi Key Laboratory of Clinical and Translational Cancer Research, Department of Oncology, The Second Affiliated Hospital of Nanchang University, Nanchang, Jiangxi China; 3 Department of Oncology, The Second Affiliated Hospital of Nanchang University, Nanchang, Jiangxi China; 4 Department of Medical Laboratory Medicine, Jiangxi Province Hospital of Integrated Chinese & Western Medicine, Nanchang, Jiangxi China; 5 Intensive Care Unit, Peking University People’s Hospital, Beijing, China; 6 Key Laboratory of Regenerative Medicine of Ministry of Education, Jinan University, Guangzhou, China; 7 Leibniz Institute on Aging - Fritz Lipmann Institute (FLI), Jena, Germany

**Keywords:** Wnt, Hematopoietic stem cells, Hematopoietic progenitor cells, DNA damage

## Abstract

Aging is characterized by the accumulation of DNA damage and a decrease in stem cell functionality, yet molecular mechanisms that limit the maintenance of stem cells in response to DNA damage remain to be delineated. Here we show in mouse models that DNA damage leads to a transient over-activation of Wnt signaling in hematopoietic stem cells (HSCs), and that high activity of canonical Wnt/β-catenin signaling sensitizes HSCs to DNA damage induced by X-irradiation which results in preferential maintenance of HSCs with low levels of Wnt signaling. The study shows that genetic or chemical activation of canonical Wnt signaling enhances radiosensitivity of HSCs while inhibition of Wnt signaling decreases it. Together, these results indicate that levels of Wnt signaling activity mediate heterogeneity in the sensitivity of HSCs to DNA damage induced depletion. These findings could be relevant for molecular alterations and selection of stem cells in the context of DNA damage accumulation during aging and cancer formation.

**Electronic supplementary material:**

The online version of this article (10.1007/s12015-019-09930-2) contains supplementary material, which is available to authorized users.

## Introduction

Functionality of adult stem cells decreases during aging [1–3], which is thought to contribute to impairments in tissue maintenance and regeneration. The underlying mechanisms involve age-dependent alterations in self-renewal pathways as well as the accumulation of DNA damage in stem cells [2, 4–7]. Possible connections between DNA damage induced aging and stem cell self-renewal pathways remain to be delineated. Loss-of-function studies show that the canonical Wnt/β-catenin signaling pathway, one of the essential self-renewal pathways, is required for the normal functioning of adult hematopoietic stem and progenitor cells (HSPCs) [8–10]. Nonetheless, the over-activation of canonical Wnt signaling was also detrimental for HSC maintenance as it causes premature exhaustion of HSCs [11–14]. Furthermore, derangement of Wnt-signaling activity contributes to impairment of stem cell function in aging tissues [15–17]. Our previous study revealed that Wnt-signaling activity determines sensitivity of intestinal stem and progenitor cells to DNA damage [18]. However, results from studies on the influence of levels of Wnt/β-catenin signaling on the maintenance of HSCs in the context of DNA damage seemed to be controversial [19, 20]. Here, we show that Wnt-signaling activity determines the sensitivity of HSPCs to DNA damage both in vitro and in vivo*,* resulting in the selection of HSPCs with low Wnt-signaling activity in the context of DNA damage.

## Materials and Methods

### Mice

C57BL/6 J mice were obtained from Hunan SJA Laboratory Animal Co. Ltd. (Hunan, China) and maintained in the animal facilities of Nanchang Royo Biotech under pathogen-free conditions on a 12-h light/12-h dark cycle. All mouse experiments were approved by the Animal Experimental Ethical Inspection of Nanchang Royo Biotech Co. Ltd. (RYEI20170913–1). All mice were 2–3 months old.

### Radiation

The radiation was performed using a commercial medical electronic linear accelerator (Varian 23EX). The samples’ position was set at SSD (source to surface distance) 100 cm from the isocenter of the machine. The radiation field size of samples was set at 20x20cm^2^. The beam used was 6MV X-ray with dose rate of 500MU/min. The daily dose output was checked using a commercial farmer ion chamber PTW 30013 which was calibrated by SSDL (secondary standard dosimetry laboratory).

### RNA Isolation, cDNA Synthesis and Quantitative Real-Time PCR

Total RNA was isolated from freshly sorted cells by using RNApure Tissue Kit (CWbiotech). TransScript-Uni One-Step gDNA Removal and cDNA Synthesis SuperMix (TransGen Biotech) was used for reverse transcriptions. qPCR was performed with an ABI 7900 Real-Time PCR System (Applied Biosystems) and TransStart Tip Green qPCR SuperMix (TransGen Biotech). Expression of genes was normalized to β–actin in each sample. Primer sets for the detection of single genes were listed in Supplementary Table S1.

### Flow Cytometry

Bone marrow cells were flushed from femurs, tibias and iliac bones, and were incubated with antibodies following standard protocols. For fluorescence activated cell sorting (FACS), all bone marrow from femurs, tibias and iliac bones were collected and all flushed cells were used. The following antibodies were used: FITC-conjugated anti-CD34 (BD Biosciences), PercpCy5.5 -conjugated anti-CD150 (BioLegend), PE-Cy7-conjugated anti-CD48 (BioLegend), APC-conjugated anti-c-Kit (BioLegend), PE-conjugated anti-Sca-1 (BioLegend) antibodies, streptavidin-APC-Cy7 (BioLegend), and lineage antibody cocktail including B220-biotin, Gr1-biotin, Ter119-biotin, CD11b-biotin, CD3-biotin, CD4-biotin, and CD8-biotin (all from BioLegend). For cell cycle analysis, Cytofix/Cytoperm Fixation/Permeabilization Solution kit (BD Biosciences) was used according to the manufacturer’s instructions. Afterwards, cells were incubated with FITC-conjugated anti-Ki67 antibody (BD) for 1 h on ice and incubated with DAPI/PBS medium to stain for DNA contents. Data acquisition and cell sorting were performed on FACS LSR Fortessa and FACS Aria III (BD Biosciences). Data were analyzed with FlowJo_V10 software.

### Cell Culture

HSPCs were cultured in StemSpan Serum-Free Expansion Medium (SFEM, StemCell Technologies) supplemented with 50 ng/ml stem cell factor (SCF; PeproTech), 50 ng/ml thrombopoietin (TPO; PeproTech), 20 ng/ml Insulin-like growth factor II (IGF-II; R&D Systems) and 10 ng/ml fibroblast growth factor 1 (FGF1; PeproTech). 6-BIO (Calbiochem) or Me-BIO (Calbiochem) and dickkopf WNT signaling pathway inhibitor 1 (DKK1; R&D Systems) were used at a final concentration of 20 nM and 500 ng/ml respectively.

### Immunostaining

Cells were sorted and stained as previously described [21]. Briefly, cells were transferred to slides (Shanghai JingAn Biological) and fixed with 4% paraformaldehyde for 10 min at room temperature (RT). Then cells were permeabilized in 0.25% Triton/PBS for 10 min at RT and blocked with 1% BSA/PBS for 1 h at RT and incubated with primary antibody Anti-phospho-Histone H2AX (Ser139) Antibody (Merck) at 1:500 dilution overnight at 4 °C. Afterwards, cells were incubated with secondary antibody anti-mouse Alexa Flour488 (Invitrogen) for 1 h at RT. To visualize the nuclei the cells were counterstained by DAPI. Images were acquired on a Leica SP5 fluorescent microscope and processed by LAS-AF-Lite_2.6.0. One hundred and fifty HSCs from 3 samples per group were scored blindly and foci were counted manually according to previously published protocols [22].

### shRNA and Lentivirus Production

The shRNA sequences were listed in Supplementary Table S2. shRNAs were cloned into SFLV-shRNA-EGFP vector using miR30 primers [23]. HEK 293 T cells were cultured in DMEM medium (Dulbecco’s Modified Eagle Medium) supplemented with 10% fetal bovine serum (FBS), penicillin (100 U/ml), and streptomycin (100 μg/ml). Lentivirus were generated in HEK 293 T cells using calcium phosphate transfection of 20 μg shRNA plasmid, 15 μg pCMVΔR8.91 helper plasmid and 6 μg pMD.G plasmid according to standard procedures [23, 24]. Culture medium was changed 12 h after transfection and virus supernatant was collected 36 h after changing medium. Virus was then concentrated by centrifugation at 25,000 rpm for 2.5 h, 4 °C, and viral pellets were responded in sterile PBS.

### Viral Transduction

3 × 10^5^ freshly sorted HSPCs (KSL cells = c-Kit^+^Sca-1^+^Lin^−^ cells) were plated in 400 μl SFEM with cytokines (see above) in a 48-well plate. Concentrated lentiviruses were added into the culture medium according to titration results. The medium was changed 12 h later. Cells were collected for further use 24 h after changing the medium.

### Transplantation Assay

Viral-transduced cells were transplanted into lethally irradiated (X-ray, 9Gy) recipient mice via tail vein injection. Recipient mice were then treated with quinolone antibiotics (enrofloxacin, Baytril) added into the drinking water (0.05%) for 1 week after transplantation and were monitored by weekly inspection until the end of the experiments. Chimerisms of GFP^+^ cells in peripheral blood from recipient mice were analyzed by flow cytometry.

### Statistics

GraphPad Prism 7.0 software was used for all statistical analysis. The unpaired two-tailed Student’s t test and One-way ANOVA were used to calculate *P* values.

## Results

### Irradiation Induces DNA Damage and a Transient Up-Regulation of Wnt Signaling in HSCs

**Fig. 1:**
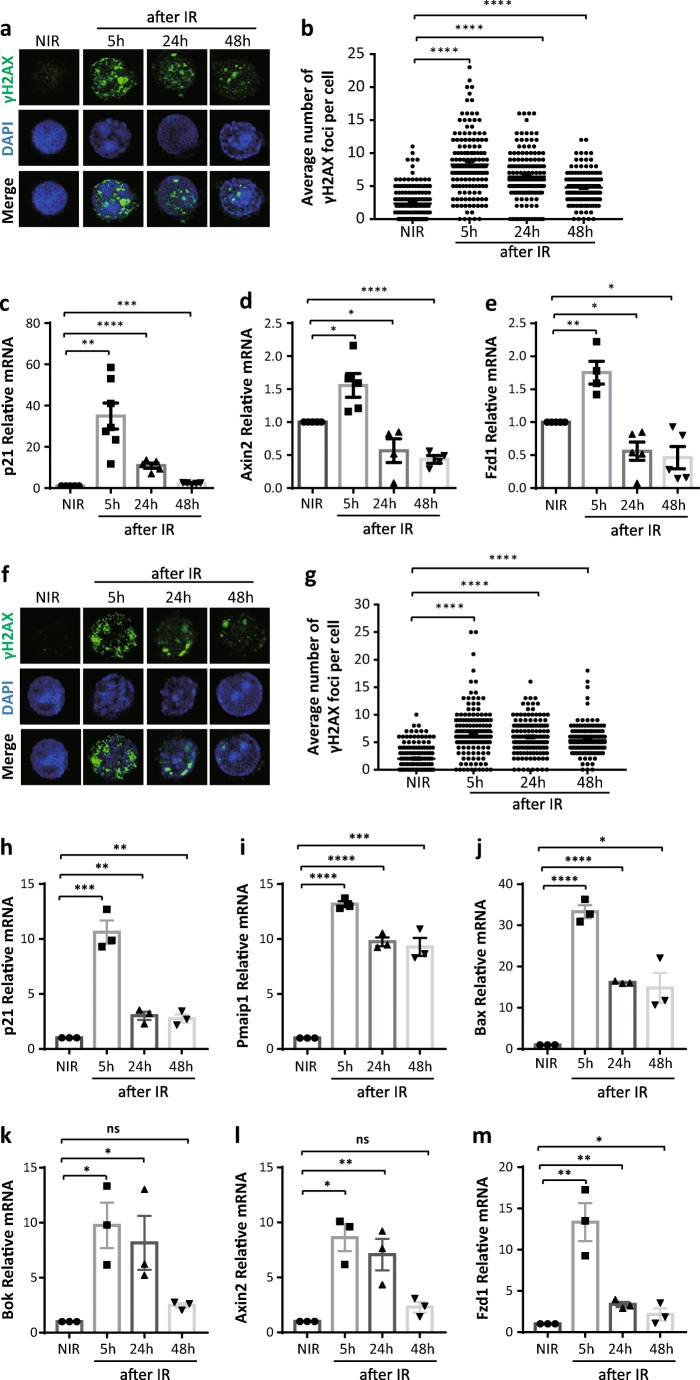
Irradiation induces a DNA damage and a transient up-regulation of Wnt signaling in HSCs (**a**, **b**) Immunostaining of p-H2AX in HSCs (CD150^+^CD34^−^ c-Kit^+^ Sca1^+^lineage^−^ cells) sorted from non-irradiated (NIR) and 2 Gy irradiated (IR) mice at indicated time points (*n* = 3 mice per group): (**a**) representative images; (**b**) statistical analysis of number of γH2AX foci (150HSCs from 3 mice were counted). (**c**–**e**) qPCR analysis of relative mRNA expression of indicated genes in HSCs sorted from non-irradiated (NIR) and 2 Gy irradiated (IR) mice at indicated time points (*n* = 4–5 mice per group). (**f**–**m**) Freshly sorted HSCs were plated in culture dishes and 2 Gy irradiated. Cells were harvested for analysis at indicated time points (n = 3 independent experiments). (**f**, **g**) Immunostaining of p-H2AX: (**f**) representative images; (**g**) statistical analysis of number of γH2AX foci (150HSCs from 3 independent experiments were counted). (**h**–**m**) qPCR analysis of relative mRNA expression of indicated genes in cultured HSCs. Results are displayed as mean ± SEM. *, *p* < 0.05; **, *p* < 0.01; ***, *p* < 0.001; ****, *p* < 0.0001; n.s., not significant; NIR, non-irradiated; IR, irradiated; h, hours

To analyze the impact of DNA damage signaling on Wnt/β-catenin signaling activity in HSCs, we irradiated young mice with 2Gy to induce an acute DNA damage. HSCs (CD150^+^CD34^−^ c-Kit^+^ Sca1^+^lineage^−^ cells) were FACS purified and analyzed for DNA damage and Wnt/β-catenin signaling kinetically after irradiation (IR). Immunofluorescent staining of p-H2AX (phospho-Histone H2AX, a classic marker of DNA damage induced by DNA double strand breaks) showed a strong induction of γH2AX foci at 5 h (5 h) that declined at later time points (24 h and 48 h) after IR (Fig. 1a, b). In addition, p21, a downstream target gene of the DNA damage signaling pathway [25], also showed a peak expression at 5 h after IR and decreased afterwards as determined by qPCR analysis (Fig. 1c). Moreover, we checked gene expression levels of Wnt components, including Axin2 and Fzd1, by qPCR analysis. Axin2 is a bona fide Wnt target gene which is commonly used for reporting Wnt signaling activity [26]. Fzd1 is a transmembrane-spanning receptor bound and activated by the Wnt ligands, thereby inducing a complex network of signaling pathways [27]. Interestingly, coinciding with a strong induction of γH2AX foci and p21, IR induced a transient up-regulation of Wnt signaling in HSCs at 5 h after IR followed by a decrease in expression levels at longer time intervals after IR (24–48 h) (Fig. 1d, e). These results indicate that irradiation triggers activation of DNA damage signaling and canonical Wnt-signaling, but that high levels of Wnt-signaling activity could not be maintained in the HSC pool in response to DNA damage.

However, as previously reported [28], we noticed a quick down-regulation of the HSC marker c-Kit post-irradiation (Fig. S1), which could result in less HSCs harvested from mice following IR when using c-Kit for HSC sorting. To investigate the effect of irradiation on DNA damage and Wnt signaling pathway in the whole population of CD150^+^CD34^−^ c-Kit^+^ Sca1^+^lineage^−^ HSCs, HSCs were purified and irradiated with 2Gy in vitro. The cells were then collected for analysis at certain time points after IR. Staining of p-H2AX and qPCR analysis also depicted similar kinetics of DNA damage and Wnt signaling activity in HSCs after IR (Fig. 1f–m). These results further support a close correlation between these two pathways after IR. Moreover, apoptotic genes, including Pmaip1 (phorbol-12-myristate-13-acetate-induced protein 1), Bax (BCL2-associated X protein), and Bok (Bcl-2-related ovarian killer), were strongly induced in HSCs which could contribute to cell death following IR (Fig. 1i–k). Notably, the expression pattern of apoptotic genes and Wnt signaling components was also closely correlated with each other (Fig. 1i–m).

It has been shown that low-level activation of canonical Wnt-signaling results in HSC expansion, whereas high-level activation of Wnt-signaling impairs HSC maintenance [29, 30]. Based on our above findings, we speculated that Wnt-signaling may increase the sensitivity of HSCs to DNA damage, thereby contributing to the elimination of HSCs in response to DNA damage.

### Modulation of Canonical Wnt/β-Catenin Signaling Activity Changes Radiosensitivity of HSPCs In Vitro

**Fig. 2:**
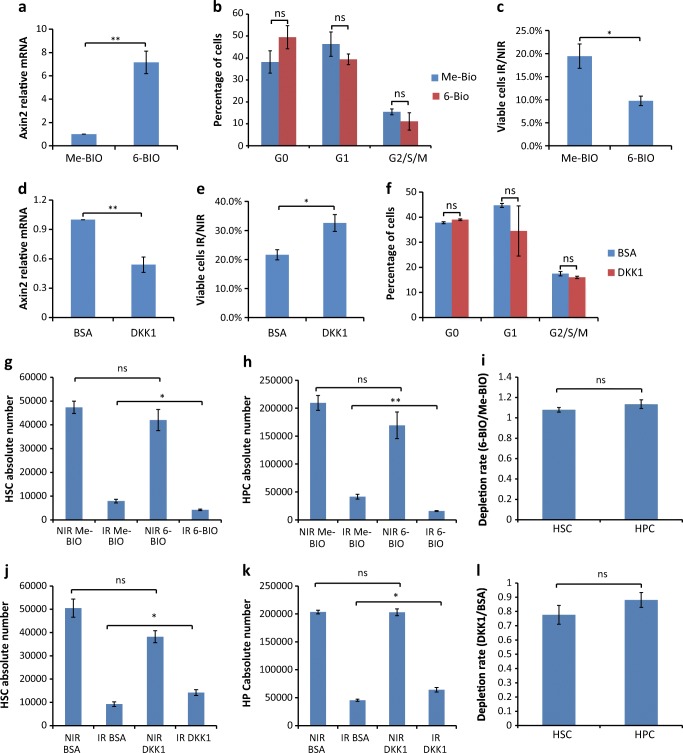
Modification of canonical Wnt/β-catenin signaling activity changes the radiosensitivity of HSPCs (**a**–**c**) 200,000 KSL cells from 2 to 3 months mice were sorted and cultured with the GSK3β inhibitor 6-BIO or with its relevant control Me-BIO for 16 h (*n* = 3 independent experiments). (**a**) Cells were collected for qPCR analysis for relative mRNA expression of Axin2 (n = 3 independent experiments). (**b**) Cell cycle analysis of cells from indicated treatment was performed on non-irradiated cells 16 h after plating. Ki67 and DAPI staining was used for flow cytometry analysis. (**c**) Treated cells were either 2 Gy X-irradiated or non-irradiated. The bar graph shows the ratio of viable cells comparing IR versus NIR of indicated treatment 24 h after IR. (**d**–**f**) 200,000 KSL cells from 2 to 3 months mice were cultured with DKK1 protein or BSA control for 16 h. (**d**) Relative mRNA expression of Axin2 was analyzed by qPCR. (**e**) Treated cells were either 2 Gy X-irradiated or non-irradiated. The bar graph shows the ratio of viable cells comparing IR versus NIR of indicated treatment 24 h after IR (n = 3 independent experiments). (**f**) Cell cycle analysis of cells from indicated treatment was performed on non-irradiated cells 16 h after plating. Ki67 and DAPI staining was used for flow cytometry analysis (n = 3 independent experiments). (**g**, **h**, **j**, **k**) Absolute number of HSCs (CD48^−^KSL cells) and HPCs (CD48^+^KSL cells) under indicated conditions determined by frequencies multiplied by absolute total cell number. (**i**, **l**) Ratios of depletion rate of HSCs (IR/NIR) versus HPCs (IR/NIR) comparing 6-BIO to Me-BIO conditions (**i**) or comparing DKK1 to BSA conditions (**l**). Results are displayed as mean ± SEM. *, *p* < 0.05; **, *p* < 0.01; n.s., not significant; NIR, non-irradiated; IR, irradiated

To test this assumption, freshly isolated KSL cells (c-Kit^+^ Sca1^+^lineage^−^ cells) were cultured in the presence or absence of a chemical activator of canonical Wnt signaling (6-BIO) and exposed to IR. 6-BIO-treatment of cultures led to an expected significant increase in Wnt-signaling activity as determined by Axin2 expression (Fig. 2a), without significantly impacting on the cell cycle activities as determined by flow cytometry analysis using a combination of Ki67 and DAPI (Fig. 2b). Interestingly, 6-BIO treatment resulted in a greater cell loss in response to IR (Fig. 2c). Together, these results revealed that activation of canonical Wnt-signaling increased the radiosensitivity of HSPCs.

To analyze whether an inhibition of Wnt would have a radioprotective effect, KSL cells were shortly exposed to the Wnt-signaling inhibitor DKK1 before IR. Compared to the BSA-treated control, DKK1-treated HSPCs possessed an expected decrease in Wnt-signaling activity (Fig. 2d), and a significant reduction in radiosensitivity (Fig. 2e). Of note, cell cycle activity was not changed by the short exposure to DKK1 (Fig. 2f) indicating that Wnt inhibition induced radioresistance in cultured HSPCs was not merely due to cell cycle inhibition.

To check whether modulation of Wnt signaling activities would impact differently on subpopulations of KSL cells, we further analyzed the composition of irradiated KSLs by combining the marker CD48 which better distinguishes HSCs (CD48^−^KSL cells) with long-term repopulating activities from the HPCs (CD48^+^KSL cells) in the culture system [31]. Flow cytometry analysis showed that CD48^−^KSL cells and CD48^+^KSL cells were depleted at similar ratios after IR, with no significant changes between Wnt-activated (6-BIO) and Wnt-inhibited (DKK1) conditions (Fig. 2g–l).

### Genetic Modification of Canonical Wnt/β-Catenin Signaling Activity Changes the Radiosensitivity of HSPCs

**Fig. 3:**
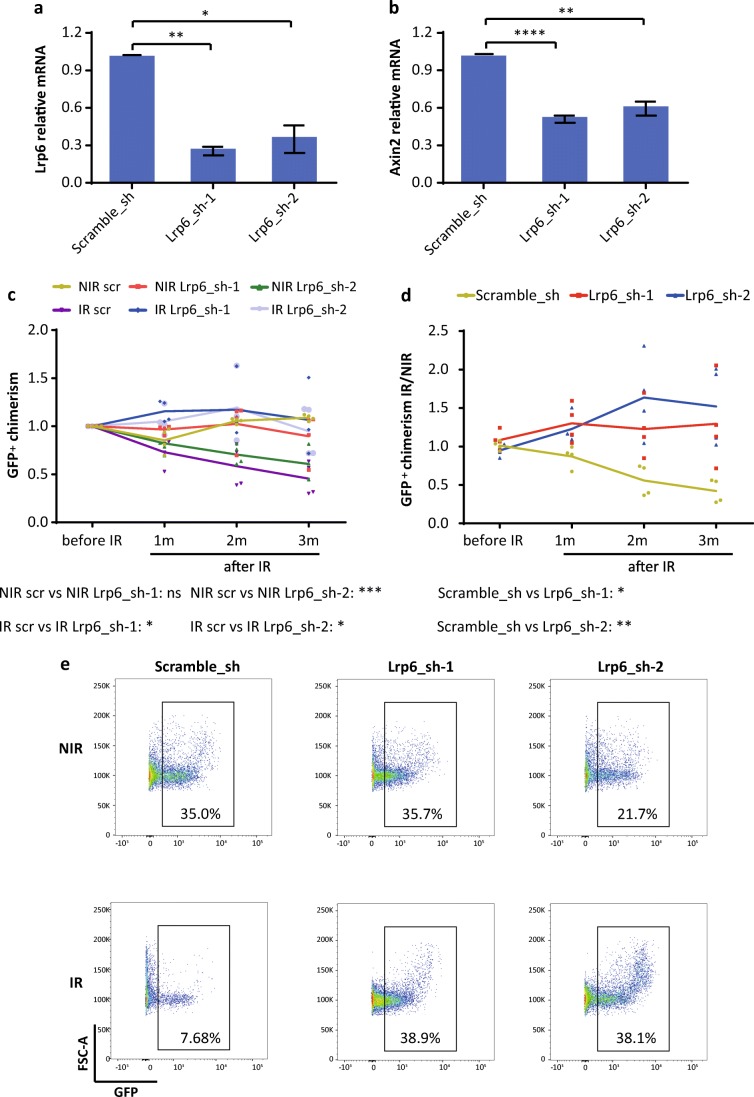
Genetic modification of canonical Wnt/β-catenin signaling activity changes the radiosensitivity of HSPCs (**a**–**e**) Freshly isolated KSL cells from 2 to 3 months mice were transduced with lentivirus expressing shRNAs targeting Lrp6, or a scramble control. For Lrp6, two different shRNAs were used. (**a**) Knockdown efficiency in KSL cells determined by qPCR of indicated shRNAs (n = 3 independent experiments). (**b**) Relative mRNA expression of Axin2 in KSL cells transduced with indicated lentivirus-shRNAs (n = 3 independent experiments). (**c**–**e**) Transduced cells (GFP^+^) were transplanted along with non-infected cells (GFP^−^) into lethally irradiated (9Gy) recipients (*n* = 8 recipients per group). Two months after transplantation, half of the recipients of each group were 4 Gy X-irradiated. The chimerism of GFP^+^ cells in peripheral blood was analyzed at indicated time points after irradiation (IR) as well as in non-irradiated controls (NIR). (C,D) GFP^+^ chimerism in peripheral blood was analyzed at indicated time points: (**c**) ratio of GFP^+^ chimerisms at indicated time points compared to the initial time point (before IR); (**d**) ratio of GFP^+^ chimerism in the irradiated group versus the mean value of the non-irradiated group. (**e**) Representative FACS plots from the analysis of peripheral blood from indicated groups 3 months after IR. Results are displayed as mean ± SEM. *, p < 0.05; **, p < 0.01; ***, *p* < 0.001; ****, *p* < 0.0001; n.s., not significant; sh, shRNA; Lrp6_sh-1, an shRNA targeting Lrp6, Lrp6_sh-2, another shRNA targeting Lrp6; NIR, non-irradiated; IR, irradiated; m, months

To test the long-term effects of Wnt-inhibition on HSPC maintenance in context with DNA damage, KSL cells were infected with lentivirus-shRNAs targeting Lrp6, a co-receptor required for activation of canonical Wnt signaling, or with a control shRNA (scramble). Two shRNAs targeting Lrp6 (Lrp6–1 and Lrp6–2) were used to avoid off-target effects. Both Lrp6-shRNAs showed around 70% knockdown efficiency (Fig. 3a), and the Wnt-signaling activity was significantly suppressed in the transduced cells (Fig. 3b). Infected (GFP^+^) KSL cells were transplanted along with residual non-infected (GFP^−^) KSL cells from the same culture into lethally irradiated recipient mice. At 2 months after transplantation, mice were split into 2 groups with one group receiving 4Gy irradiation and the other not. GFP^+^ chimerism in peripheral blood was monitored for up to 3 months following IR. The ratio of GFP^+^ chimerism (normalized to condition before IR) in these mice revealed both Lrp6 hairpins maintained a similar level of GFP^+^ chimerism over 3 months when irradiated, which sharply fell in the scramble control (Fig. 3c). Additionally, in NIR conditions the Lrp6–2 hairpin showed a decreasing GFP^+^ chimerism over time, which is lost upon irradiation. When the ratio of GFP^+^ chimerism in the irradiated group was normalized to the mean of the non-irradiated group (Fig. 3d) one can more clearly see the protective effects of Lrp6 knockdown, with again both hairpins maintaining the level of GFP^+^ chimerism over 3 months when irradiated whilst the scramble control drops to roughly 50%. This can also be observed in the representative peripheral blood FACS plots at 3 months after IR (Fig. 3e).

Together these data indicate that down-regulation of Wnt signaling via knockdown of Lrp6 benefits the long-term maintenance of HSPCs with DNA damage, though the long-term repopulating activities might be impaired under NIR condition.

## Discussion

The current study provides experimental evidence that the level of canonical Wnt-signaling activity mediates the sensitivity of HSPCs to DNA damage. These results are in line with previous studies showing that Wnt-signaling activity was elevated upon irradiation induced DNA damage in HSCs and can amplify DNA damage responses in other cell types [32–34]. The studies provide a plausible explanation for the dual effects of Wnt signaling on HSPC maintenance and elimination depending on the level and duration of Wnt-signaling in HSPCs.

In the current study, 2Gy were used for the in vitro experiment, and for the in vivo experiments, both 2Gy and 4Gy were used. In vitro irradiation dose (2Gy) was assumed to be roughly equivalent to in vivo experiments (2Gy) and 2-fold less than the in vivo experiments (4Gy). This assumption was based on the mice and cell culture dishes being of similar mass and size and irradiated with identical doses. Both 2Gy and 4Gy were used to achieve induction of DNA damage in the current study, which is unlike the case of radiation therapy where the prescribed dose can be derived from equations such as BED (biologically effective dose) and EQD2 (equivalent dose in 2Gy fractions), with parameters of fraction numbers, dose per fraction, the ratio of α/β etc. Since both 2Gy and 4Gy are relatively low irradiation doses that are within the sublethal dose range of mice, we would expect to observe similar results under both conditions in this study.

A wealth of studies has proved that the Wnt signaling pathway is essential for self-renewal and regeneration activities upon injury of the ISCs [35–37], which supports the hypothesis that instructed enhancement of Wnt signaling would benefit intestinal regeneration after irradiation. For instance, it was shown that activation of Wnt signaling resulted in less apoptosis and enhanced proliferation after IR in the gastrointestinal system [38, 39]. However, Tao et al. have also shown that niche positioning determines the Wnt signaling activity of intestinal stem and progenitor cells (ISPCs), with ISPCs at the intestinal crypt bottom exhibiting higher Wnt/−catenin activity than the ISPCs located at higher positions of the crypt. ISPCs with higher Wnt signaling activity are preferentially depleted by irradiation-induced DNA damage [18]. Furthermore, instructed enhancement of Wnt signaling increases radio-sensitivity of ISPCs, while inhibition of Wnt signaling decreases it [18]. These seemingly contradictory results from different studies on the role of Wnt signaling activity on fate and activities of ISPCs upon DNA damage might due to the different angles of the studies: Tao et al. particularly looked at the early fate (i.e. survival) of ISPCs upon DNA damage and different subpopulations of ISPCs with intrinsic different Wnt signaling activities, while the other mentioned studies looked at the reconstitution activities of ISPCs at later time points after irradiation [38, 39]. Our current study showing that up-regulation of Wnt signaling activity sensitizes HSCs to irradiation was in line with the study from Tao et al. One might speculate that HSCs with higher Wnt activity are more similar to ISCs located at crypt bases with higher Wnt signaling activity. Of note, ISCs are highly proliferative cells, whereas the majority of HSCs reside in deep quiescence. It would be difficult to directly translate results between two completely different systems. For example, Ascl2 (achaete scute-like 2, a Wnt target gene) together with β–catenin/Tcf, activates the genes fundamental to the intestinal stem cell state [40]. Wnt signaling has been proved to be essential for in vitro culture of intestinal stem cells [41, 42]. However, in the hematopoietic system, genetic knockout or inhibition of Wnt essential components leading to down-regulation of Wnt/β–catenin signaling is dispensable for differentiation and self-renewal of adult murine hematopoietic stem cells as well as for hematopoietic homeostasis [43, 44].

Results from previous studies on how Wnt signaling influences DNA damage and hematopoietic regeneration after irradiation have also been controversial. Lento et al. have shown that constitutive depletion of β-catenin led to impaired regeneration of the hematopoietic system, accumulation of reactive oxygen species (ROS), and inability to repair DNA damage at later time points after irradiation [19]. However, a more recent study by Himburg et al. showed that inhibition of Wnt-signaling with DKK1 increased the recovery of HSPCs of irradiated mice [20]. These contradictory studies suggested that the effect of Wnt signaling on DNA damage was very complicated. Notably, genetic depletion of β-catenin might have different effect on the level of Wnt signaling compared to acute modification by proteins, small molecules or shRNA knockdown, such as DKK1 and 6-BIO. In line with the study by Himburg et al., our study showed that Wnt-signaling was activated at an early time point in response to DNA damage in HSPCs. We further proved that HSPCs with an intrinsically lower level of Wnt signaling activity, achieved by shRNA knock-down of LRP6, exhibited advantages in competition during regeneration of the hematopoietic system after DNA damage. Moreover, we showed that the instructed activation of Wnt-signaling by 6-BIO treatment resulted in greater loss of HSPCs while inhibition of Wnt-signaling by DKK1 improved the survival of HSPCs after irradiation. These results suggest that Wnt-activation is a part of feed-forward-loop contributing to the depletion of HSPCs in context with DNA damage. The study therefore extends the current understanding of the role of Wnt signaling in early fate decision and survival of HSPCs upon irradiation induced DNA damage.

Preferential maintenance of HSPCs with low Wnt-signaling activity in response to DNA damage could influence clonal drifts and the selection of aberrant stem cells during aging and carcinogenesis. Although over-activation of Wnt-signaling leads to exhaustion of non-transformed stem cells [12–14, 18], activation of Wnt-signaling has been frequently observed in tumors [45–47]. One possible explanation is that cancer cells may inactivate DNA damage checkpoints (such as p53) to escape Wnt dependent amplification of the DNA damage response. Supporting this argumentation, it was shown that tumors induced by elevated Wnt-signaling were more progressive upon p53 deficiency [48–50] . Together, the current study could have important implications for understanding HSC aging and carcinogenesis involving the accumulation of genetic alterations and the clonal selection of stem cells.

## Electronic supplementary material

Supplementary Figure 1. c-Kit expression was down-regulated after irradiation. Representative FACS plots of bone marrow cells collected from mice under indicated conditions. (PDF 1624 kb)

ESM 2 (DOCX 19 kb)
