# Supplementary figures and images for: High Canonical Wnt/β-Catenin Activity Sensitizes Murine Hematopoietic Stem and Progenitor Cells to DNA Damage
Source: Stem Cell Rev Rep. 2019 Dec 3;16(1):212–21. doi: 10.1007/s12015-019-09930-2 (PMC6987068; doi:10.1007/s12015-019-09930-2)

**NIR**

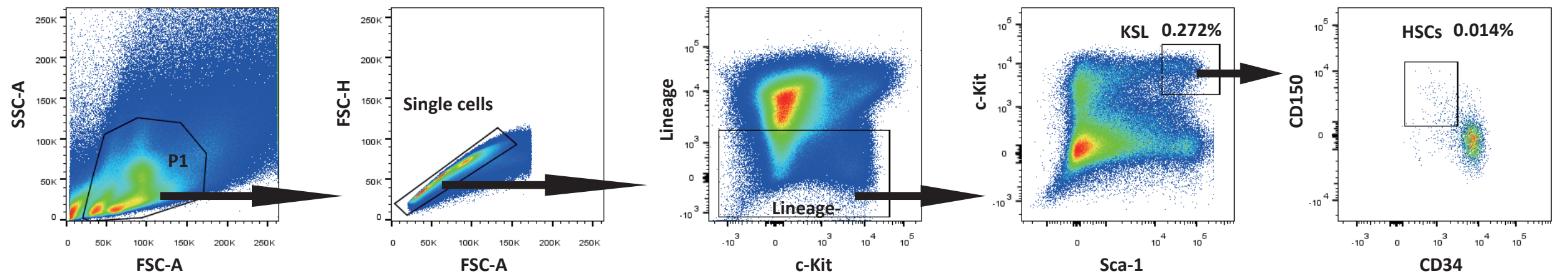

**IR 5h**

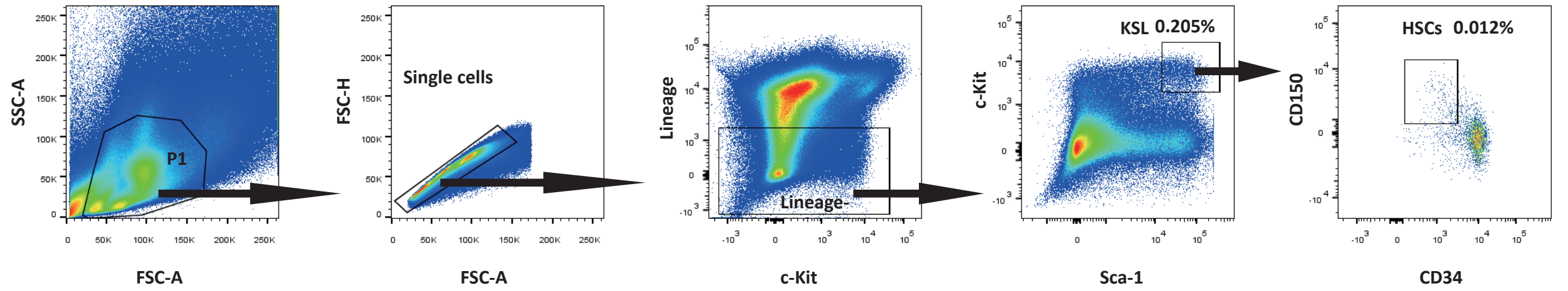

**IR 24h**

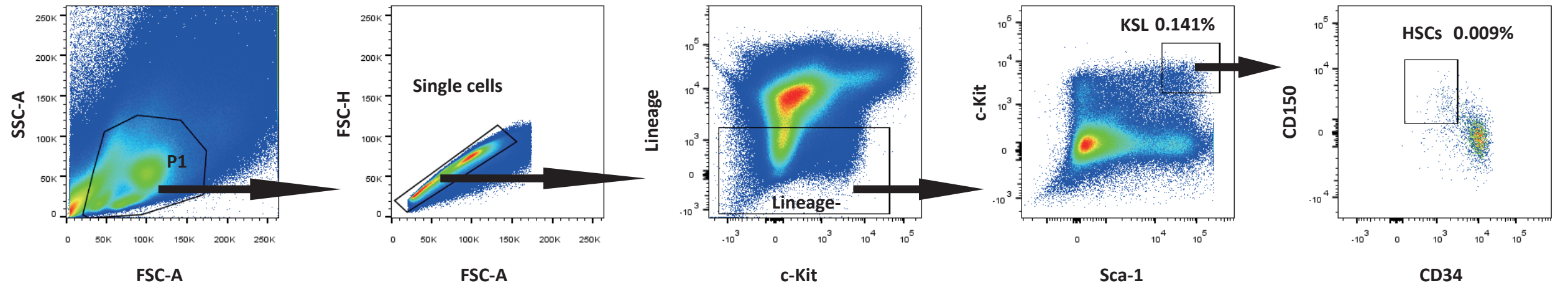

**IR 48h**

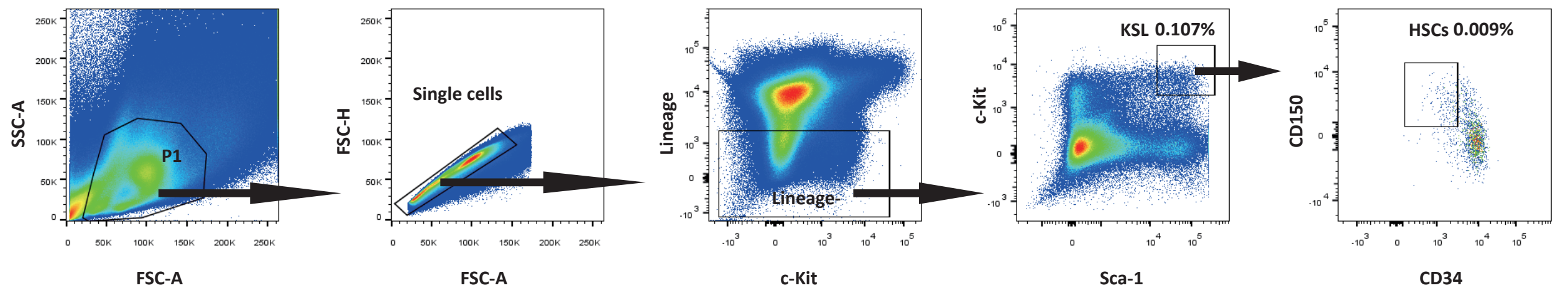

Supplement: Supplementary file 1 — c-Kit expression was down-regulated after irradiation. Representative FACS plots of bone marrow cells collected from mice under indicated conditions. (PDF 1624 kb) [file 12015_2019_9930_MOESM1_ESM.pdf]
