# Supplementary material for: High Canonical Wnt/β-Catenin Activity Sensitizes Murine Hematopoietic Stem and Progenitor Cells to DNA Damage
Source: Stem Cell Rev Rep. 2019 Dec 3;16(1):212–21. doi: 10.1007/s12015-019-09930-2 (PMC6987068; doi:10.1007/s12015-019-09930-2)
Supplement: Supplementary file 2 — (DOCX 19 kb) [file 12015_2019_9930_MOESM2_ESM.docx]

**Table S1.Primer list**

| **Gene** | **Forward Primers** | **Reverse Primers** |
| --- | --- | --- |
| β-actin | CTAAGGCCAACCGTGAAAAG | ACCAGAGGCATACAGGGACA |
| Axin2 | GAGAGTGAGCGGCAGAGC | CGGCTGACTCGTTCTCCT |
| Cdkn1a(p21) | AACATCTCAGGGCCGAAA | TGCGCTTGGAGTGATAGAAA |
| Fzd1 | CAGCAGTACAACGGCGAAC | GAGATGGGCTGGCAGTAGC |
| Lrp6 | TGCAAACAGACGGGACTTGAG | CGGGGACAATAATCCAGAAACAA |
| Pmaip1 | CAGATGCCTGGGAAGTCG | TGAGCACACTCGTCCTTCAA |
| Bax | GTGAGCGGCTGCTTGTCT | GGTCCCGAAGTAGGAGAGGA |
| Bok | AGTGGCAGGCCACATCTT | CCACGGAATACAGGGACACTA |

**Table S2. Short hairpin sequences**

| **shRNA symbol** | **shRNA sequence** |
| --- | --- |
| scrambled control | CGCAGTTCGATATCTACTGAAA |
| Lrp6_shRNA-1 | CCTGGACAGATTCTGAAACTAA |
| Lrp6_shRNA-2 | CCTGGACTGATATATCACTGAA |
